# Omecamtiv mecarbil lowers the contractile deficit in a mouse model of nebulin-based nemaline myopathy

**DOI:** 10.1371/journal.pone.0224467

**Published:** 2019-11-13

**Authors:** Johan Lindqvist, Eun-Jeong Lee, Esmat Karimi, Justin Kolb, Henk Granzier

**Affiliations:** Department of Cellular and Molecular Medicine, University of Arizona, Tucson, Arizona, United States of America; Cinvestav-IPN, MEXICO

## Abstract

Nemaline myopathy (NEM) is a congenital neuromuscular disorder primarily caused by nebulin gene (NEB) mutations. NEM is characterized by muscle weakness for which currently no treatments exist. In NEM patients a predominance of type I fibers has been found. Thus, therapeutic options targeting type I fibers could be highly beneficial for NEM patients. Because type I muscle fibers express the same myosin isoform as cardiac muscle (Myh7), the effect of omecamtiv mecarbil (OM), a small molecule activator of Myh7, was studied in a nebulin-based NEM mouse model (*Neb* cKO). Skinned single fibers were activated by exogenous calcium and force was measured at a wide range of calcium concentrations. Maximal specific force of type I fibers was much less in fibers from *Neb* cKO animals and calcium sensitivity of permeabilized single fibers was reduced (pCa_50_ 6.12 ±0.08 (cKO) vs 6.36 ±0.08 (CON)). OM increased the calcium sensitivity of type I single muscle fibers. The greatest effect occurred in type I fibers from *Neb* cKO muscle where OM restored the calcium sensitivity to that of the control type I fibers. Forces at submaximal activation levels (pCa 6.0–6.5) were significantly increased in *Neb* cKO fibers (~50%) but remained below that of control fibers. OM also increased isometric force and power during isotonic shortening of intact whole soleus muscle of *Neb* cKO mice, with the largest effects at physiological stimulation frequencies. We conclude that OM has the potential to improve the quality of life of NEM patients by increasing the force of type I fibers at submaximal activation levels.

## Introduction

Nebulin is a giant filamentous protein, located in the skeletal muscle sarcomere[1] where it winds around the actin filament, from the Z-disk to near the pointed end of the actin filament[2]. Nebulin is believed to play an important role in muscle contraction[3–5], it maintains myofibrillar alignment[6] and functions as a thin filament stabilizer that regulates thin filament length[7]. Nebulin deficiency due to mutations in the nebulin gene (NEB) is an important cause of Nemaline Myopathy (NEM)[8–11]. Although the phenotypes of nebulin-related NEM patients are variable, a common functional feature is skeletal muscle weakness [12–17]. No treatments for NEM exist to improve muscle function. In NEM patients a predominance of type I fibers has been found [13, 16]. Thus, therapeutic options targeting type I fibers would be highly beneficial for NEM patients.

Omecamtiv mecarbil (OM) is a selective small-molecule activator of cardiac myosin (Myh7 or β-MHC) that was developed as a treatment for heart failure [18, 19]. Single molecule studies have shown recently that OM suppresses the size of the working stroke of myosin and prolongs the duration of its attachment to actin [20]. It has been proposed that OM-inhibited myosin heads cooperatively activate the thin filament at submaximal activation levels, recruiting OM-free myosin heads and increasing force [20].

**Fig 1:**
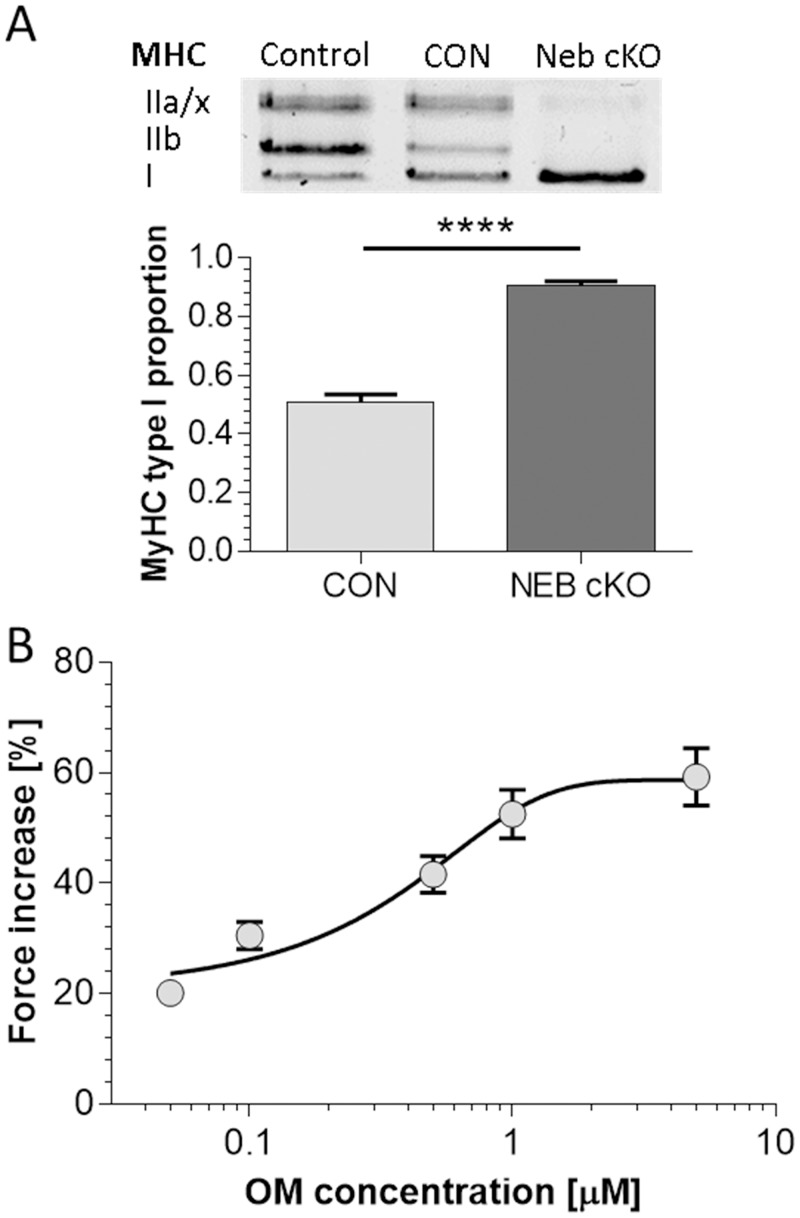
Fiber type composition of *Neb* cKO muscle and dose-response curve of OM. (A) The proportion of type I myosin heavy chain (MHC) isoforms. (Top) Typical MHC-gel of soleus muscle of six months old CON and cKO mice. Control muscle (left lane) is a mixture of 40 days old TC and Sol muscle to reveal all MHC isoforms. In CON, MHC type I and IIa/x isoforms are present, while only the MHC type I isoform is clearly detectable in Neb cKO muscle. Bottom: The proportion of type I MHC is significantly greater (****: p<0.0001) in Neb cKO (n = 25) than CON (n = 20) mice. (B) Dose-response of OM on soleus muscle from *Neb* cKO mice (n = 6). Forces were measured at a 20Hz stimulation frequency. Relative increases in force from vehicle are shown. Relative increases in force beyond vehicle are shown. Results follow a dose-response curve with EC_50_ of 0.47±0.08 μM (based on fitting results from each individual muscle). Values are mean ± SEM.

Although OM has been developed for heart failure patients, cardiac myosin (Myh7 or β-Myh) is identical to the type I myosin expressed by slow skeletal muscles[21] suggesting that OM might also be effective in skeletal muscle. Furthermore, type I fibers are the dominant fiber type in most human muscles [22, 23]. The percent type I fibers in healthy humans is >60% for a wide range of muscle types [23] and examples of type I rich muscles in the mouse are the piriformis (~60%), the quadratus femoris (~70%)[24], and the soleus (~50%, Fig 1). Furthermore, studies have shown that in both mouse models of NEM and in NEM patients there is an additional shift towards type I fibers [12–15, 25]. Thus, OM might be effective in increasing skeletal muscle force, particularly in NEM patients. We investigated whether OM has a beneficial effect on slow skeletal muscle and used a conditional nebulin knockout mouse model (*Neb* cKO) which expresses low levels of nebulin and has severe weakness in peripheral and respiratory muscles[25], phenocopying NEM[9, 16, 25, 26]. Additionally, there is a fiber type I predominance [25], as in NEM patients. Hence, we tested the hypothesis that OM ameliorates the force deficit in *Neb* cKO mice.

## Materials and methods

The conditional nebulin knockout (*Neb* cKO) mouse was previously described [25]. Mice that were positive for MCK-Cre and homozygous for the floxed nebulin allele were nebulin deficient and are referred to as *Neb* cKO. Littermate controls (CON) were WT for nebulin[25]. Studies were performed on membrane-permeabilized soleus muscle fibers and intact whole soleus muscles. Six months old *Neb* cKO (189±3 days old) and CON (186±1 days old) mice were used for this study. In this work we focused on the fiber type I rich soleus muscle. The weight of the soleus muscle was 7.7 ± 0.5 mg (n = 20) in CON and 11.6 ± 0.7 mg (n = 25) in Neb cKO mice and the physiological cross-sectional area at mid-belly was 0.58 ± 0.03 mm^2^ (CON) and 0.91 ± 0.05 mm^2^ (*Neb* cKO). This is similar to our previous study [25] in which it was found that most muscles in the *Neb* cKO mice are atrophied, with as exception the soleus muscle that hypertrophies. Furthermore, it is known from previous work that the fiber cross-sectional area of type I fibers in the soleus is reduced significantly in the *Neb* cKO while the number of type I fibers is greatly increased [25]. All experiments were in accordance with the United States Public Health Service’s Policy on Humane Care and Use of Laboratory Animals and approved by the University of Arizona Institutional Animal Care and Use Committee.

### Omecamtiv mecarbil

Omecamtiv mecarbil (OM) was purchased from Selleckchem (Houston, TX) and was dissolved in dimethylsulfoxide (DMSO) to make a stock solution as instructed by the manufacturer. OM stock solution was then added to the experimental solution to prepare the final desired concentration of OM. In most studies, we used a low OM dose (0.1 μM) to limit effects on cardiac muscle that occur mainly at OM concentration > 0.3 μM [18, 19, 27–30].

### Skinned muscle fiber mechanics

#### Experimental procedure

**Fig 2:**
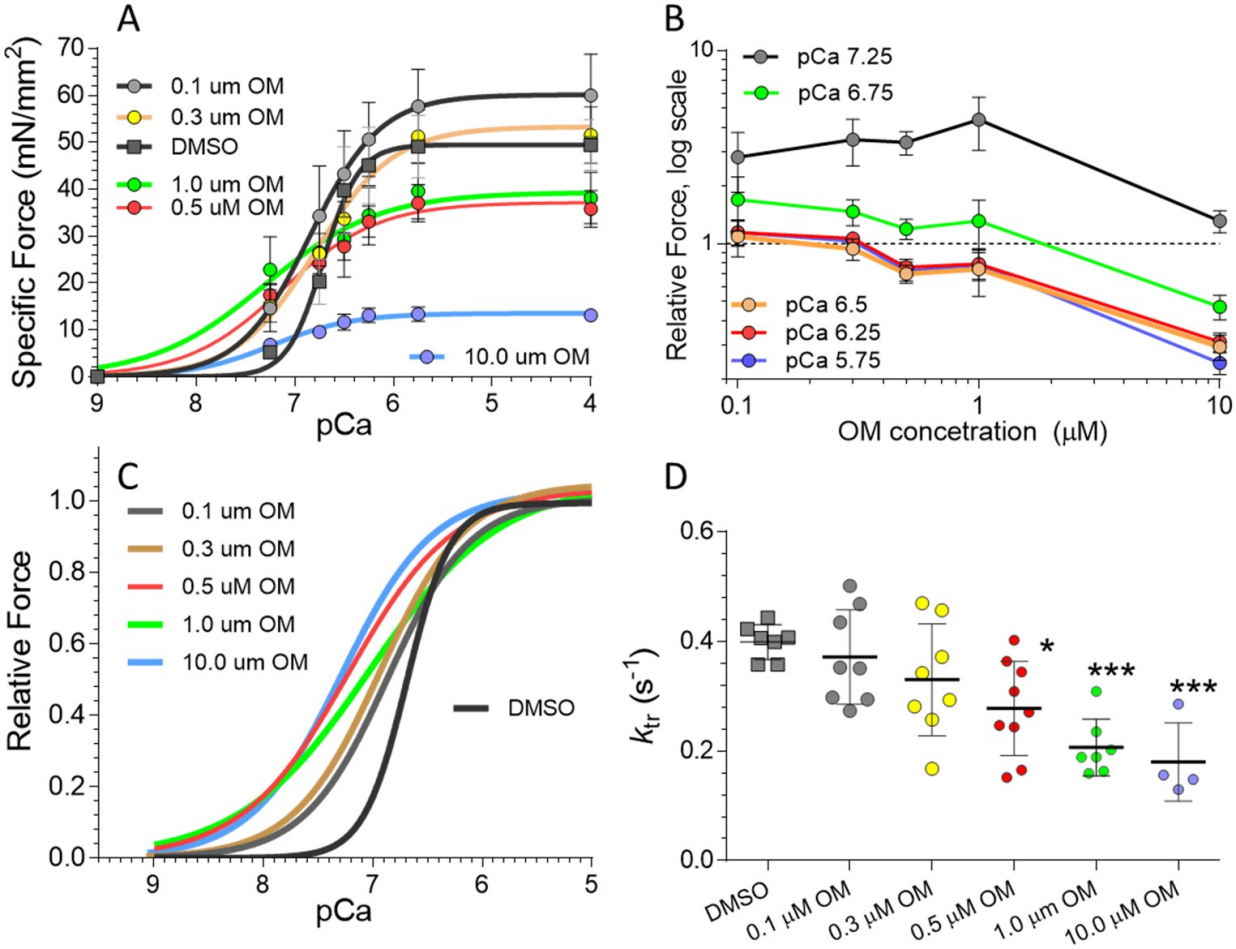
Effect of OM on force-pCa relations of permeabilized fiber bundles from Neb cKO mice. A) Force-pCa curves in 0, 0.1, 0.3, 0.5, 1.0 and 10.0 μM OM. OM affects specific force in a dose-and activation-dependent manner. B) Force in OM relative to 0.0 OM at 5 pCa values. The two lowest OM concentrations (0.1 and 0.3 μM OM) increase force at low activation levels with minimal effects at high activation. At 0.5, 1.0, and 10 μM OM forces are minimally increased at low activation level and forces at high activation levels are depressed. C) Force-pCa curves normalized to their maximal force (pCa 4.0) are left shifted in OM (increased pCa50) and less steep (reduced Hill coefficient). D) Ktr values (pCa 6.75) at a range of OM concentrations. At high OM doses, Ktr is reduced. A and B shows mean ± SEM and C shows curve fits of data from 7 muscles except for the DMSO (vehicle) and 10 μM OM data that represent 6 muscles each. In D each data point represents one muscle. Asterisks in D denote significant differences between vehicle and OM (1W ANOVA with multiple testing correction); one symbol p<0.05 and three symbols p<0.001.

**Fig 3:**
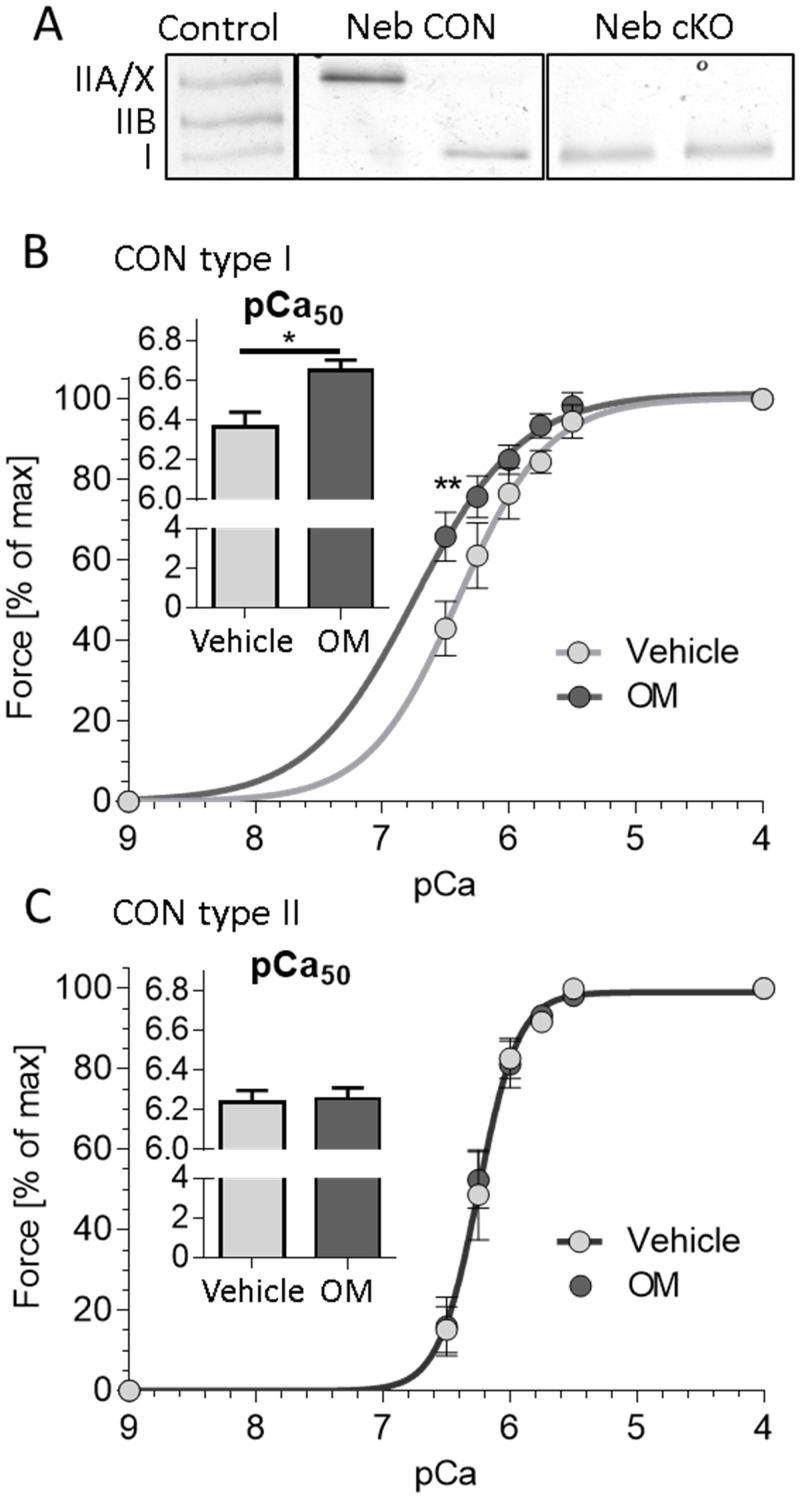
Effect of OM on active force of single soleus fibers from CON mice. A) Representative images of silver-stained gel with single fiber lysates loaded. Control is a mix of soleus and EDL lysates that shows the resolvable MHC isoforms (IIX and IIA integrate into a single band). Neb CON shows one type IIX/A fiber and one type I fiber while Neb cKO displays two type I fibers. (B and C) Relative force-pCa curves for slow type I (B) and fast type II (C) muscle fibers from CON soleus mice. Inset in B: Calcium-sensitivity (pCa_50_) of slow type I fibers with 0.1 μM OM was significantly greater than fibers with vehicle. Values are mean ± SEM. Multiple fibers per animal were studied (typically 5–6 fibers per animal) and their means were calculated. The shown values are the means of all studied animals: 5 mice with total 29 fibers (vehicle) and 5 mice with 22 fibers (OM) for type II fibers and 5 mice with 28 fibers (vehicle) and 5 mice with 25 fibers (OM) for type I fibers. (*p<0.05; **p<0.01 comparison vehicle vs. OM).

**Fig 4:**
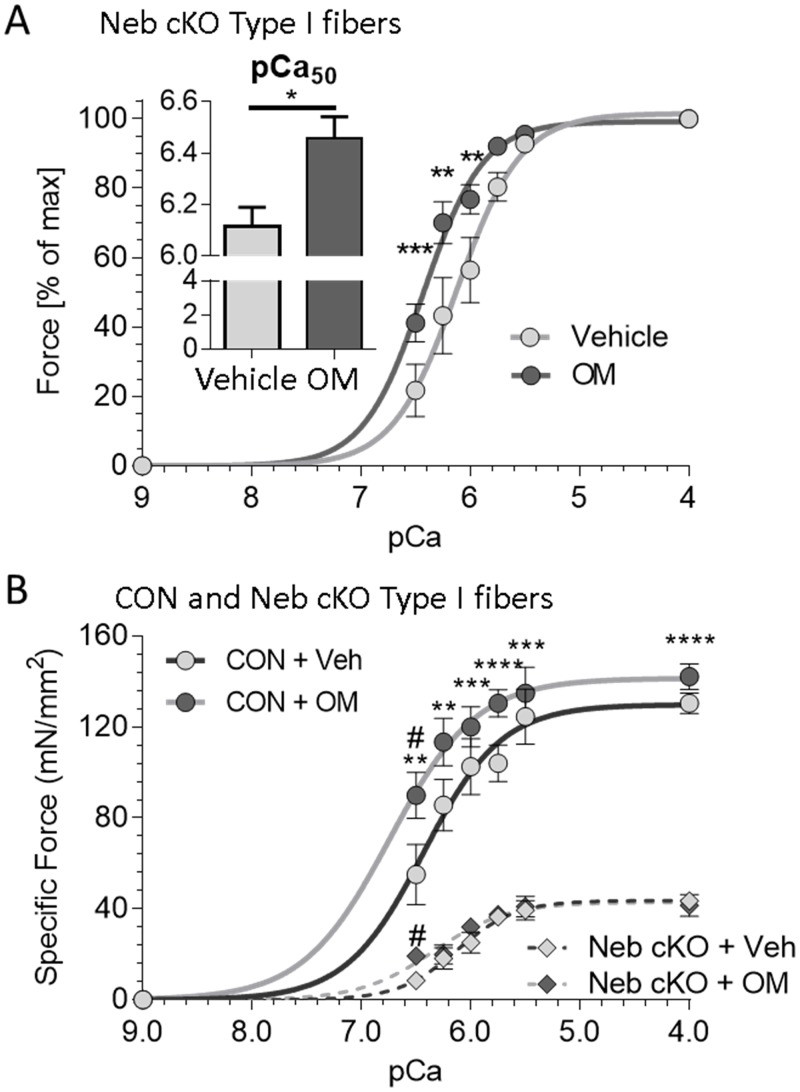
Effect of OM on active force of single type I fibers of *Neb* cKO and comparison with CON type I fibers. (A) Relative force-pCa relations of single type I fibers from *Neb* cKO soleus muscle. Relative forces were significantly greater at pCa 6.50, 6.25 and 6.00 with OM in *Neb* cKO fibers. Inset: Calcium sensitivity (pCa_50_) was significantly greater in fibers with OM than with vehicle. (B) Specific force-pCa relations in CON (solid lines) and cKO (dashed lines) in type I fibers with vehicle (Veh) or OM. Force-pCa curves were left shifted with OM relative to vehicle in both genotypes. Values are mean ± SEM. Multiple fibers per animal were studied and their means were calculated. The shown values are the means of 5 mice with total 28 fibers (CON + vehicle), 5 mice with 25 fibers (CON + OM) for type I fibers and 6 mice with 40 fibers (cKO+vehicle) and 5 mice with 28 fibers (cKO+OM). (*p<0.05; **p<0.01; ***p<0.001: ****p<0.0001; comparison CON vs. cKO, ^#^p<0.0001 comparison vehicle vs. OM).

The procedures for skinned muscle mechanics were as described previously[31]. Briefly, soleus muscles were skinned overnight at ∼4°C in relaxing solution (in mM: 40 BES, 10 EGTA, 6.56 MgCl_2_, 5.88 NaATP, 1 DTT, 46.35 K-propionate, 15 creatine phosphate, Ionic strength 180 mM, pH 7.0 at 20°C) containing 1% (w/v) Triton X-100 and protease inhibitors (in mM: 0.01 E64, 0.04 leupeptin and 0.5 PMSF). Muscles were then washed thoroughly with relaxing solution and stored in 50% glycerol/relaxing solution at −20°C[32]. Either fiber bundles (Fig 2) or single fibers (Figs 3 and 4) were dissected and mounted using aluminum T clips between a length motor (ASI 322C, Aurora Scientific Inc.), and a force transducer element (ASI 403A, Aurora Scientific Inc.) in a skinned fiber apparatus (ASI 802D, Aurora Scientific Inc.). Sarcomere length was set in passive fibers to 2.4 μm using a high-speed camera and video-based sarcomere length software (ASI 900B, Aurora Scientific Inc.); in a small subset of control fibers we measured sarcomere length and found no difference in sarcomere shortening between OM treatment vs. vehicle (DMSO). Muscle bundles/fibers were activated in pCa (pCa = –log([Ca^2+^]) 4.0 activating solution (in mM: 40 BES, 10 CaCO_3_ EGTA, 6.29 MgCl_2_, 6.12 Na-ATP, 1 DTT, 45.3 potassium-propionate, 15 creatine phosphate, Ionic strength 180 mM, pH 7.0 at 20°C) and protease inhibitors. Fiber width and depth (built-in prisms allow for side view of fibers and measurement of depth) were measured at three points along the fiber, and the cross-sectional area (CSA) was calculated assuming an elliptical cross-section. The CSA of type I fibers was on average 1300 μm^2^ for CON and 1183 μm^2^ for *Neb* cKO fibers. Specific force was expressed as force per CSA (mN/mm^2^) and used for comparison of force between groups. In a separate series of experiments we also used skinned cardiac muscle strips (LV papillary)[33] and performed OM dose-response experiments (pCa 6.0) as explained above for skeletal muscle, except that the sarcomere length to which the passive cardiac muscle was set prior to activation was 2.0 μm.

### Protocols

Preparations were in relaxing solution and then immersed in pre-activating solution (relaxing solution with a 10-fold lower EGTA concentration), followed by activation with incrementally increased pCa (pCa = –log([Ca^2+^]), ranging from 6.5 to 4.0. Solutions with different pCa were created by mixing varying volumes of relaxing and activation solutions. The obtained force–pCa relation was fit with a Hill equation, providing pCa_50_ (pCa giving 50% of maximal active force) and the Hill coefficient, *n*_H_, an index of myofilament cooperativity. Experiments were performed at 20°C. Separate groups of muscle bundles/fibers were used to test the effect of vehicle (DMSO) and OM (in DMSO). Fibers from a total of five CON and five *Neb* cKO mice, with multiple fibers (4–6) per animal were used in fiber experiments. Five to seven soleus bundles from two *Neb* cKO were used for dose-response studies. *k*_tr_-measurements: The rate of tension redevelopment (ktr) was measured at steady-state force by rapidly shortening (1 ms) the fiber at one end of the fiber resulting in unloaded shortening of the fiber for 20 ms. Remaining bound cross-bridges were detached by rapidly restretching the fiber to initial length and the tension redeveloped [34]. ktr was determined by fitting the rise of force to the following equation (one-phase association curve): F = Fss*(1−ektr*t)+c, where F is force at time t, Fss is steady-state force. After mechanical experiments, the bundles/fibers were stored in SDS-sample buffer for gel electrophoresis and myosin isotyping (see below).

### Intact muscle mechanics

#### Muscle experiments

Mice were anesthetized with isoflurane and sacrificed by cervical dislocation. The soleus muscle was dissected and mounted to the isolated muscle test system (1200A, Aurora Scientific Inc.) that has been described previously[35]. In brief, both tendons were tied with 5–0 silk sutures, and the muscle was attached between the lever arm of dual servomotor-force transducer (300C, Aurora Scientific Inc.) and a fixed hook. The experimental bath was filled with Krebs-Ringer solution containing (in mM) 137 NaCl, 5 KCl, 1 MgSO_4_, 2 CaCl_2_, 1 NaH_2_PO_4_, 24 NaHCO_3_, 11 Glucose with 95% O_2_ and 5% CO_2_ supply (pH = 7.4). The temperature during the experiments was 30°C. The muscle was placed between platinum electrodes connected to an electrical stimulator (701C, Aurora Scientific Inc.) for muscle activation. The optimal muscle length (L_0_) was determined by adjusting overall muscle length until maximal force was generated with a 400ms 20Hz stimulation frequency (pulse duration of 200μs with biphasic polarity). The muscle length at L_0_ was measured using a digital caliper. At L_0_, an initial tetanic contraction at 150Hz stimulation frequency was imposed. Measured forces were normalized to the physiological cross-sectional area (PCSA) to obtain specific force (mN/mm^2^). PCSA (in mm^2^) was calculated using muscle mass (in g), fiber length (in mm), and muscle density (1.056 g/mm^3^);PCSA = Muscle mass / (Fiber length × Muscle density). Since muscle fibers of soleus align to their tendon with a pennation angle and do not span the full muscle length, fiber length is estimated from muscle length and pennation angle. As reported in Burkholder et al.[36], the ratio of fiber and muscle length for soleus is 0.72. We used this ratio to calculate the fiber length from the measured muscle length.

#### Force-frequency protocol

To establish the force-frequency relation, active forces at various stimulation frequencies were measured. 14 CON and 14 *Neb* cKO mice were used for the force-frequency protocol. The muscle was stimulated at 1, 5, 10, 20, 30, 40, 60, 100 and 150 Hz and waiting for 30, 30, 60, 90, 90, 120, 120, 180 and 180 s, respectively, in between stimulations. The first force-frequency protocol was performed in the vehicle solution (DMSO), and the experimental solution was then switched to OM solution. The muscle was incubated in OM solution for 15 min with twitch stimulation every minute to monitor changes in force. When the increase in force was stable, the force-frequency protocol was measured.

#### Force-velocity protocol

To determine the force-velocity relation, the load-clamp technique was used as previously described[37]. Shortening velocities were measured during isotonic contraction against loads 85, 70, 55, 40, 25, 15, and 5% of maximal force at 25 Hz of stimulation frequency. Each contraction started out with a 200 ms isometric phase (to reach the plateau force), and then a 150 ms load-clamp was applied. A series of load-clamps was performed in solution with vehicle and the same protocol was then repeated in OM solution. The muscle was incubated in OM solution for 15 min with twitch stimulation every minute before the protocol with OM solution was performed. After completion of each experiment, the sutures and tendons were carefully removed, and the muscle mass was then measured. The muscle was quick-frozen in liquid nitrogen and stored at −80°C for later analysis of fiber type composition.

#### Analysis

The force-frequency curve was fit with a Hill equation to calculate the half-maximal frequency (frequency giving 50% of maximal force increase beyond twitch force) and the Hill coefficient (*n*_*H*_), an index of the steepness of the curve. Shortening velocity (in mm/sec) was measured as a slope of linear portion of the shortening period (~40 ms duration) after force was stable. For each preparation, shortening velocity was normalized by muscle fiber length. Muscle power production was calculated by multiplying the shortening velocity and the applied load. Force-power curves were fitted by 2^nd^ order polynomial non-linear regression, and the maximal power production was determined from the apex of the force-power curve.

## Myosin heavy chain isoform determination and distribution

Sodium dodecyl sulfate polyacrylamide gel electrophoresis was used to determine the myosin isoform composition of the muscle lysates and single fibers as previously described [35]. The stacking gel contained a 4% acrylamide concentration (pH 6.7), and the separating gel contained 8% acrylamide (pH 8.7) with 30% glycerol (v/v). The gels were run for 24 h at 15 °C and a constant voltage of 275 V. Gels for whole muscle lysates were stained with Coomassie blue and single fiber gels were silver-stained. Gels were scanned and analyzed with ImageJ (v1.49, NIH, USA).

## Statistical analyses

**Fig 5:**
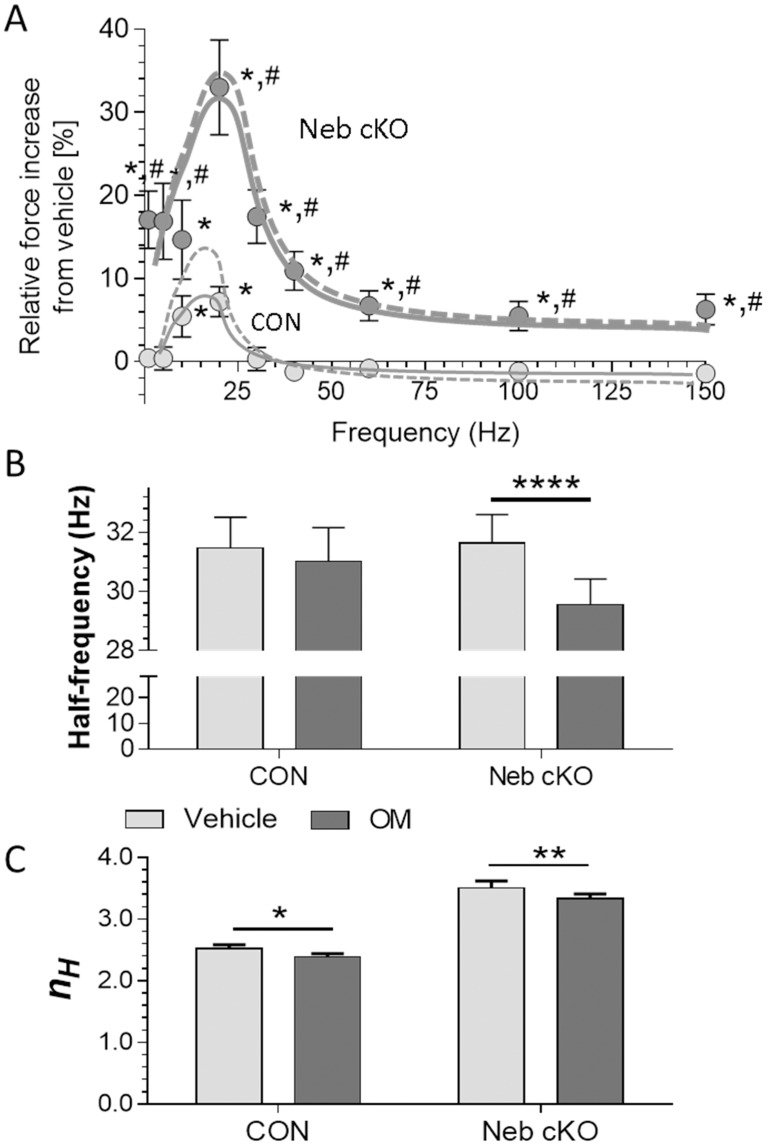
Effect of OM on force of intact soleus muscle. (A) Relative increase in force as a function of frequency in OM over vehicle. *Neb* cKO muscle (dark symbols); CON (light symbols). The relative increase in force with OM over vehicle was increased at all stimulation frequencies in *Neb* cKO muscles, while significance was found at only 10 and 20Hz in CON muscle. The OM effect was significantly greater in *Neb* cKO than CON at all frequencies except 10 Hz. The solid lines are the fits to the data and the broken lines normalize the response to the slow type I myosin content (50% in control muscle and 90% in *Neb* cKO muscle (Fig 1A)). (B-C) frequency for half-maximal force (half-frequency) in (B) and Hill-coefficient in (C) with vehicle or OM. A two-way repeated measures ANOVA revealed in B) a significant treatment effect with a significant interaction with genotype and in C) a significant treatment effect, a significant genotype effect and an interaction between treatment and genotype. Sidak multiple comparison tests revealed in B) a significant OM effect in *Neb* cKO muscle and in C) a significant OM effect in both CON and *Neb* cKO muscles. The number of muscles is 14 for each genotype. Values are mean ± SEM. Asterisk(*) comparison of OM vs. vehicle; sharp(#) comparison cKO vs. CON. One through four symbols: p<0.05, p<0.01, p<0.001, and p<0.0001, respectively.

**Fig 6:**
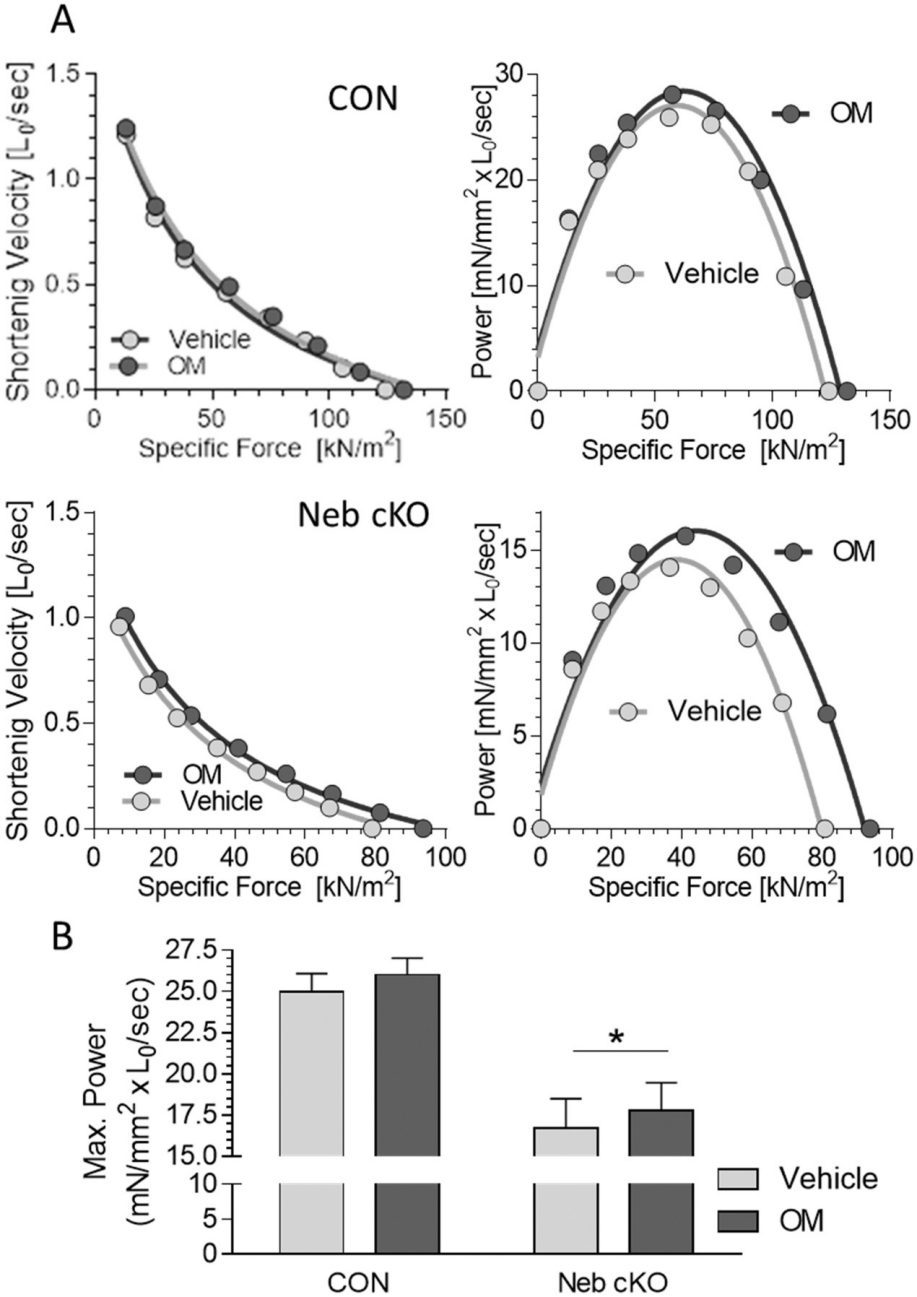
Effect of OM on power production in intact soleus muscle. A) Examples of force-velocity (left) and force-power curves (right) in CON (top) and *Neb* cKO (bottom) in vehicle and OM. (B) Maximal power with vehicle and OM solutions in both genotypes. A two-way repeated measures ANOVA revealed a significant treatment effect and a significant genotype effect. A Sidak multiple comparison test revealed a significant OM effect in *Neb* cKO muscle. *Neb* cKO mice have decreased power compared to CON. OM-treatment increased power in *Neb* cKO mice. Numbers of muscles are 6 for CON and 11 for cKO. Values are mean ± SEM. Comparison between vehicle and OM is marked as an asterisk(*). One symbol p<0.05.

Data are presented as mean ± SEM. GraphPad Prism 6 was used to calculate statistics. For statistical analysis one-way ANOVA, two-way ANOVA and the t-test with multiple testing corrections were used, as appropriate. For permeabilized single fibers study, Student’s t-tests were performed on force-pCa curve (each pCa) and pCa_50_ of each fiber type and genotype (OM vs. Vehicle; Figs 3 & 4). For the force-frequency curve of the intact whole muscle experiment, paired t-tests were used at each stimulation frequency (OM vs. Vehicle). Student’s t-tests were also used for comparison of genotype (CON vs. cKO) in MHC type I proportion (Fig 1A) and relative force increase (Fig 5A) in intact whole muscle experiment. Repeated measure 2-way ANOVA and multiple post-hoc t-test using Sidak method were performed for half-frequency, Hill-coefficient (Fig 5B and 5C), and maximal power (Fig 6B) analysis. p < 0.05 was considered to be statistically significant.

## Results

### Fiber type composition of *Neb* cKO muscle and Dose-response of OM

It has been previously reported that nebulin deficiency leads to a shift in myosin heavy chain isoform distribution from fast type II to slow type I MHC[25]. In order to test this in the mice studied here, SDS-PAGE was performed on lysates obtained from muscles used in the mechanical studies. Representative MHC gels and analyzed results are shown in Fig 1A. The average proportion of type I MHC isoform in soleus muscle was 51±3% in control (CON) muscle and 91±1% in *Neb* cKO muscle. Thus the soleus muscle of *Neb* cKO muscle expresses predominately type I myosin. We also performed studies to establish the OM dosage for our experiments and measured the OM effect in intact *Neb* cKO soleus muscle activated with 20 Hz stimulation. As shown in Fig 1B, OM significantly increased force; this effect was seen already at 0.05 μM OM and followed a dose-response curve with an EC_50_ of 0.6 μM.

The OM dose-response curve was also established for skinned fiber bundles dissected from soleus muscle of *Neb* cKO mice. (Note that these fiber bundles are fiber-type mixed and that studies with fiber-typed single fibers are described later in the Results section). Fiber bundles were sub-maximally activated by exogenous calcium at a range of OM concentrations (S1A Fig). All tested OM concentrations increased force production. Interestingly, the force increase reaches a plateau between 0.5 μM and 1.0 μM, and declined at a higher dose (S1A Fig). The half-maximal effect occurred at ~0.1 μM (the force decline at 10 μM precluded fitting the data to a standard dose-response curve). For a comparison we also performed experiments on submaximally-activated permeabilized left ventricular papillary muscle and found that force increased more gradually and did not decline at high OM doses (S1B Fig, see also Discussion).

We also determined the OM effect on the full force-pCa relationship of soleus fiber bundles. At 0.1 and 0.3 μM OM, force was increased, particularly at submaximal activation levels (Fig 2A and 2B). At 0.5 μM and higher the force increase at submaximal activation was small and at maximal activation, force was depressed (Fig 2B). Note that force depression was absent in the intact muscle experiments (Fig 1), most likely because these experiments were performed at a frequency (20 Hz) that produces submaximal force levels (see also Discussion). Normalized force-pCa curves of soleus fiber bundles, with force normalized to the maximal force (pCa 4.0), are left shifted in OM (increased pCa_50_) and less steep (reduced Hill coefficient), Fig 2C. We also investigated cross-bridge cycling kinetics by measuring the rate constant of tension redevelopment (*k*_tr_) in *Neb* cKO bundles at a range of OM-concentrations. Starting at 0.5 μM OM, *k*_tr_ is decreased (Fig 2D). As *k*_tr_ correlates with myosin ATPase activity [34, 38], these experiments suggest that high OM doses slow ATPase activity. Based on these results and the desire to limit the likelihood of OM effects on cardiac muscle [18, 27, 28, 30] (see also Discussion), 0.1 μM OM was used in a follow-up study focused on single fibers that have been fiber-typed.

### Force measurements on fiber-typed single fibers

The effect of OM on contractility was first studied by measuring the Ca^2+^ dependence of force in membrane-permeabilized soleus slow type I and fast type II single fibers. This comparative study was performed only on CON fibers (that express nebulin) because of the limited number of type II fibers in *Neb* cKO soleus muscle (out of the 50 studied fibers only four fibers (one treated with vehicle and three treated with OM) were type II, which precluded statistical testing). Examples of single fiber gels are shown in Fig 3A. Single fibers were activated with incrementally increasing concentrations of Ca^2+^ with or without 0.1 μM OM. Treatment of fibers with OM resulted in a robust increase in submaximal force production in type I fibers (Fig 3B). We did not observe any effect of OM on type II fibers (Fig 3C). The results in type I fibers show an OM-induced increase in pCa_50_ (the pCa for half-maximal force) of 0.29 pCa-units (insets of Fig 3B and 3C). Thus, at a submaximal activation level, OM significantly affects force production of type I fibers.

Soleus type I fibers from *Neb* cKO mice were studied next. Compared to type I fibers from CON muscle these fibers had significantly lower maximal specific force and significantly lower calcium sensitivity (Table 1), as shown by their reduced pCa_50_ (6.12 in cKO and 6.36 in CON). The low specific-force levels of the *Neb* cKO fibers are likely due to the absence of nebulin but the role of other adaptive changes cannot be excluded. It is known that at the whole muscle level profound changes in expression occur in MHC, Troponin-T, Troponin-I and Tropomyosin isoforms [25]. This is likely due to the known fiber-type switch from fast to slow, but determining whether differences exist when comparing the same fiber type in different genotypes will require future work focused on single fiber proteomics. OM had no effect on the maximal active force, but at submaximal Ca^2+^ levels, forces were significantly increased (Fig 4A). The greatest increase was at low Ca^2+^ levels, the effect became less as Ca^2+^ was increased and was absent at maximal activation (Fig 4A). At intermediate calcium levels the increase was large, for example at pCa 6.5, OM increased specific force from 8.4±2.6 mN/mm^2^ to 19.2±3.5 mN/mm^2^. However, it is important to point out that although specific force was more than double; it still was much below that of untreated control fibers (55 mN/ mm^2^ at pCa 6.5).

**Table 1:** Effect of OM on active force of single fibers (top) and intact soleus muscle (bottom).

**Single fibers**	**Max. Specific force (mM/mm^2^)**		**pCa_50_**			***n*_H_**		
	Veh	OM	Veh	OM	Δ	Veh	OM	Δ
CON type II	131.4±5.5	146.3±8.1	6.20±0.06	6.23±0.04	0.03±0.01	3.66±0.11	3.26±0.44	0.54±0.33
CON type I	130.5±4.56	144.1±5.60	6.36±0.08	6.65±0.05 (*)	0.29±0.10	1.81±0.35	1.54±0.33	0.28±0.27
*Neb* cKO type I	43.6±3.16 (####)	41.6±4.88 (####)	6.12±0.10 (#)	6.46±0.08 (*)	0.34±0.04	2.10±0.31	1.84±0.52	0.36±0.62
**Whole muscle**	**Max. Specific force (mM/mm**^**2**^**)**		**Freq. for 50% of maximal force (Hz)**			***n***_**H**_		
	Veh	OM	Veh	OM	Δ	Veh	OM	Δ
CON	297.8±15.1	294.1±15.7	31.5±1.0	31.0±1.1	0.45±0.27	2.52±0.06	2.39±0.05(*)	0.13±0.02
*Neb* cKO	118.6±9.4 (####)	124.6±8.9 (***,####)	31.6±1.0	29.5±0.8 (****)	2.10±0.27(###)	3.50±0.11 (####)	3.33±0.07 (*,####)	0.17±0.06

(*) comparison between OM and Veh (#) comparison between *Neb* cKO and CON

The OM-induced increase in force at sub-maximal activations in type I *Neb* cKO fibers resulted in a leftward shift of the force-pCa curves (Fig 4A). The Hill coefficient (*n*_H_), a measurement of the filament cooperativity, was also determined. Although mean *n*_*H*_ values were reduced by OM, the differences were not significant (Table 1). The OM-induced leftward shift of the force- pCa relation increased the pCa_50_ from 6.12±0.10 to 6.46±0.08 in *Neb* cKO fibers (Fig 4A) which is close to that of type I CON fibers in vehicle (pCa 6.36, Table 1).

### Effect of OM on intact muscle

Intact soleus muscles were electrically stimulated at a range of frequencies to determine their force-frequency relation. The maximal specific force of *Neb* cKO soleus was ~1/3 of the maximal specific force of CON soleus, consistent with the maximally activated single fiber data (Table 1) and highlighting that the force deficit of intact *Neb* cKO muscle has a myofilament origin. OM has a small but significant effect on force at all stimulation frequencies. The increase was maximal at 20Hz (32±5%) and gradually decreased at higher stimulation frequencies (Fig 5A, dark symbols). However, in CON muscle the effect was less (Fig 5A, light gray symbols) and only reached significance at 10 Hz (increase 5±2%) and 20Hz (increase 7±2%). From the force-frequency curves, the frequency for half-maximal force increase beyond twitch force (defined as half-frequency) and the Hill coefficient (*n*_H_) were calculated (Fig 5B and 5C). The average half-frequency was decreased by OM but this effect was only significant in *Neb* cKO muscle (Fig 5B and Table 1). Hill coefficients were significantly decreased by OM in both genotypes, and the effect was largest in cKO muscle (Table 1).

The shortening velocity at a range of load levels was also measured. The shortening velocity was normalized by muscle length, and the force-velocity curves were converted to force-power curves and the effects of OM were studied. Fig 6A show example experiments and 6B show the mean results of all experiments. OM had no significant effect on CON muscles but it enhanced the maximal power in *Neb* cKO muscles, the effect was small but significant (Fig 6B).

## Discussion

Nemaline myopathy (NEM) is the most common non-dystrophic congenital myopathies, with features that include muscle weakness, muscle atrophy, presence of nemaline rod-bodies and type I fiber predominance[8, 9, 12–17]. It impacts both peripheral and respiratory muscles, and many NEM patients have difficulties in locomotion and respiratory function[17]. However, targeted treatment options for NEM patients are not available. Although OM has been developed as a heart failure drug, the expression of cardiac myosin (Myh7 or β-MHC) in both heart and slow skeletal muscle[21], predicts that OM is effective in slow skeletal muscle as well, and the findings of the present study clearly bear this out. OM greatly increases the force of single type I fibers activated at submaximal activation levels and significantly increases the calcium sensitivity, as reflected by the increased pCa_50_ (Fig 4). Considering the large number of type I fibers in humans [22–24] and the additional shift in fiber type distribution of nebulin-deficient muscle and NEM patients toward type I fibers [12–15], OM might be a therapeutic option to ameliorate the muscle weakness in NEM patients. That OM is well-tolerated and is currently in phase-3 clinical trials for treatment of heart failure makes it an attractive therapeutic option. Our findings suggest that OM is far from a cure. However, it has a good potential to bring relief in nebulin-based nemaline myopathy patients, not only because of their type I predominance but also because nebulin deficiency appears to increase the effectiveness of OM. Below we discuss our findings in detail.

OM has been developed to increase the contractile force of cardiac muscle in heart failure patients[18]. Unlike various existing calcium sensitizers[39] OM does not affect calcium transients, which limits possible side effects (e.g., altering calcium-dependent signaling processes) [18, 40]. Recent single molecule studies [20] provide evidence that OM binds myosin, causing long-lived strongly bound myosin heads that do not generate force. From these studies, a model was proposed in which these OM-bound heads cooperatively activate the thin filament at submaximal activation levels, recruiting OM-free myosin heads and thereby increasing force [20]. This mechanism naturally explains the sarcomere length dependence of the magnitude of the OM-effect that has been found in sub-maximally activated cardiac muscle[41], with larger OM effects at short sarcomere length where the baseline activation level of the thin filament is lower and more OM-free heads can be recruited to the force generating pool than at longer sarcomere length. It can also explain the reduced OM effects at high OM levels reported by Nagy et al. for both cardiac myocytes and diaphragm fibers[42], and seen here in soleus fiber bundles (S1A Fig): at high OM levels the OM-bound heads that do not generate force outnumber the additionally recruited OM-free force generating heads. The dose-response curve that we measured on cardiac muscle does not decline at high OM levels (S1B Fig) a finding similar to that of Gollapudi et al (their Fig 2)[41]. This can be explained by the short sarcomere length that was used in these studies (2.0 μm in our study and 1.9 μm in [41]), where baseline activation is low, and the long sarcomere length used by Nagy et al in their cardiac myocyte study (2.3 μm) where the baseline activation level is high. Thus a mechanism based on an increased attachment lifetime of OM bound non-force producing myosin heads that enhances thin filament activation can explain the various findings. An additional mechanism recently proposed by Kampourakis et al. [43] to explain the OM effect involves the ON and OFF states of the thick filament [44, 45]. In the OFF state, many of the myosin heads in the thick filament are bent back towards the center of the sarcomere and are unable to interact with actin and in the ON state they are in a more perpendicular position and available for actin binding and force generation. By promoting the ON state of the thick filament and increasing the thin filament activation level, OM might facilitate actomyosin interaction and enhance force development at low to intermediate calcium levels [43, 45, 46]. The extent to which both thick filament and thin filament based mechanisms are responsible for OM-induced force increase requires future research.

The effects that were found in type I fibers (increased pCa_50_-values and no effect on the maximal force) are in general similar to the OM effects reported in cardiac muscle. In cardiac muscle of mouse[47], rat[42, 43], guinea pig[41] and human[28], OM increases calcium sensitivity (higher pCa_50_), and typically has no effect on the maximal active force. Our findings are also consistent with the work of Nagy et al who recently used OM on rat diaphragm muscle fibers[42]. Although the myosin isoform was not directly determined, Nagy et al did measure the kinetics of force generation (*K*_*tr*_) which also provides insights in the fiber type (slow kinetics for type I fibers and fast kinetics for type II fibers). In both their and our study, OM clearly increases the calcium sensitivity of slow type I fibers (fibers with low *K*_*tr*_ values in Nagy et al[42] and Myh7 in our study). The Nagy et al study also found a small but significant effect of OM on type II fibers (identified by Nagy et al as having fast kinetics[42]), which we did not observe. This issue warrants follow-up. If future studies were to show that OM also affects type II MHC isoforms, it would extend OM’s usefulness for treating skeletal muscle diseases with as it would increase the proportion of fibers being affected by OM. It would also represent a treatment option for myopathies with fiber type II dominance.

Most cardiac studies were performed at OM levels higher than ours (0.3–1.0 μM), except for studies on guinea pig myocardium[41] and human myocardium[28] (both of which found no effect at 0.1 μM) and a study on rat myocytes [45]that did find an effect at 0.1 μM but that was smaller than in the present study (e.g., ΔpCa_50_ ~0.1, our study ~0.3). Thus it appears that the sensitivity to OM is higher in our study on type I skeletal muscle fibers than in published studies on cardiac muscle. The explanation for this is not immediately clear but could reside in the distinct protein isoform composition of thin and thick filament proteins involved in activation in slow skeletal muscle compared to cardiac muscle. It is also possible that the tissue-specific expression of nebulin (present in skeletal muscle but absent in cardiac muscle[48]) is part of the explanation, or that some other putative adaptation in the proteome of nebulin-free skeletal muscle fibers play a role. Future work is required to fully understand the details of the OM effect on both cardiac and skeletal muscle. The higher OM sensitivity of skeletal muscle type I fibers, compared to cardiac muscle, is beneficial as it provides a therapeutic window for a sole skeletal muscle effect. For OM to be useful for increasing skeletal muscle force in nebulin-based nemaline myopathy patients, undesirable effects in the heart have to be avoided. Slowing of force relaxation in the heart is of a particular concern since this can impair diastolic filling and cause ischemia, especially at high heart rates. However, clinical trials have shown that OM is well tolerated without adverse effects up to ~10 μM in healthy individuals, and in heart failure patients at plasma concentrations of up to ~1.0 μM (reviewed in[39]). Thus it is possible that the doses established in the present study will be able to increase skeletal muscle force in Nemaline Myopathy patients without adverse effects on the heart. Finally, it is also worth pointing out that when using OM in heart failure patients, undesirable effects on skeletal muscle have to be avoided. This could occur, for example, if high OM doses required for cardiac benefits in heart failure patients were to suppress force production or crossbridge cycling kinetics of type I fibers of the diaphragm. The possible clinical application of OM requires study of both the cardiac and skeletal muscle systems.

The effect of OM on intact muscle reached a maximum at 20Hz and then gradually decreased at higher stimulation frequencies (Fig 5A). This is similar to the findings in single fibers and, thus, in both preparations, the greatest effect of OM occurs at submaximal activation levels. Because skeletal muscles operate at submaximal activation levels during normal activity [22, 49, 50], OM is expected to enhance force development under clinically meaningful conditions. OM does not nearly recover the large functional deficit of nebulin deficient muscle and, thus, OM is far from ‘a cure.’ Considering that our work was performed at a low OM concentration (0.1 μM), which is below the EC_50_ of 0.6 μM, it is likely that the effect of OM can be augmented by increasing the OM dose. From our present study it is possible to conclude OM is likely to provide relief to NEM patients as increases in force of ~20% are clearly attainable, which is likely to improve quality of life for patients with muscle weakness.

It is also worth noting that OM has a small effect on intact muscle of *Neb* cKO mice at simulation frequencies that result in maximal tetanic force (Fig 5A), and that this is not the case in single fibers that are maximally activated (pCa 4.0) (Fig 4B, Table 1). This suggests that unlike in maximally activated skinned fibers, in intact muscle of *Neb* cKO mice, the number of myosin molecules participating in maximal tetanic contraction is not at its maximal level. This conclusion is consistent with an X-ray study on intact muscle that revealed that, compared to CON muscle, *Neb* cKO muscle has thick filaments that partially remain in the OFF state during a maximal tetanus [51]. Our findings indicate that OM corrects this effect by more completely switching the thick filament to its ON state. This mechanism (a deficit in the ON state of the thick filament in the absence of nebulin) can also explain the larger effect of OM on type I fibers of *Neb* cKO muscle compared to CON type I fibers. Finally, it also provides an explanation for the only study in which OM was found to increase the force of maximally activate skinned cardiac muscle (pCa ~4.0)[41]. This finding was obtained at a short sarcomere length (1.9 μm) where the thick filament might not be fully turned ON with calcium[52], and, thus, OM is able to augment the ON state of the thick filament and increase maximal force.

In summary, this study shows for the first time that at submaximal activation levels OM increases force and power of slow skeletal muscles. This is expected to be beneficial for nemaline myopathy patients where type I fibers dominate, with predicted benefits for respiratory and peripheral muscle function. OM currently is in a Phase 3 Heart Failure clinical trial (GALACTIC-HF). The previous phase I and phase II clinical trials have shown that OM is well tolerated to plasma levels of ~1.0 μM [39], a level beyond the level used in the present study. We conclude that OM has the potential to improve skeletal muscle function in NEM patients and bring much needed relief. Finally, it is also worth considering that although type II fibers do not appear to benefit from OM, fast skeletal muscle troponin activators (FASTA) have been developed that selectively activate type II fibers by increasing their sensitivity to calcium [53]. Indeed it has been shown that FASTAs increase force at submaximal activation levels in type II skeletal muscle fibers from nebulin-deficient mice and nemaline myopathy patients [54, 55]. Thus, by using a combination of both OM and FASTA, an increase in force of all fiber types might be achievable.

## Supporting information

S1 Fig A) Dose-response of OM effect on force production by soleus fiber bundles from Neb cKO mice (n = 6). Specific force was measured at pCa 6.75. OM increases specific force at low OM doses, the effect is maximal between 0.5 and 1.0 μM OM, and is much less at 10 μM OM. B) OM Dose-response using LV permeabilized papillary muscle from control (n = 7) and Neb cKO mice (n = 7). Specific force was measured at pCa 6.0. Specific force follows a dose-response curve with EC50 of 0.62±0.04 μM OM (control) and 0.79±0.08 μM OM (Neb cKO). No significant difference in EC50 (See text for details).
(TIF)

## References

[1] Wang K. Purification of titin and nebulin. Methods Enzymol. 1982;85 Pt B:264–74. 10.1016/0076-6879(82)85025-8 . 6896900

[2] Labeit S, Kolmerer B. The complete primary structure of human nebulin and its correlation to muscle structure. J Mol Biol. 1995;248(2):308–15. 10.1016/s0022-2836(95)80052-2 . 7739042

[3] Chandra M, Mamidi R, Ford S, Hidalgo C, Witt C, Ottenheijm C, et al Nebulin alters cross-bridge cycling kinetics and increases thin filament activation: a novel mechanism for increasing tension and reducing tension cost. J Biol Chem. 2009;284(45):30889–96. 10.1074/jbc.M109.049718 19736309 PMC2781488

[4] Ottenheijm CA, Granzier H. Lifting the nebula: novel insights into skeletal muscle contractility. Physiology (Bethesda). 2010;25(5):304–10. 10.1152/physiol.00016.2010 . 20940435

[5] Bang ML, Caremani M, Brunello E, Littlefield R, Lieber RL, Chen J, et al Nebulin plays a direct role in promoting strong actin-myosin interactions. FASEB J. 2009;23(12):4117–25. 10.1096/fj.09-137729 19679637 PMC2812046

[6] Tonino P, Pappas CT, Hudson BD, Labeit S, Gregorio CC, Granzier H. Reduced myofibrillar connectivity and increased Z-disk width in nebulin-deficient skeletal muscle. J Cell Sci. 2010;123(Pt 3):384–91. 10.1242/jcs.042234 20053633 PMC2816184

[7] Pappas CT, Krieg PA, Gregorio CC. Nebulin regulates actin filament lengths by a stabilization mechanism. J Cell Biol. 2010;189(5):859–70. 10.1083/jcb.201001043 20498015 PMC2878950

[8] Sanoudou D, Beggs AH. Clinical and genetic heterogeneity in nemaline myopathy—a disease of skeletal muscle thin filaments. Trends Mol Med. 2001;7(8):362–8. 10.1016/s1471-4914(01)02089-5 . 11516997

[9] Lawlor MW, Ottenheijm CA, Lehtokari VL, Cho K, Pelin K, Wallgren-Pettersson C, et al Novel mutations in NEB cause abnormal nebulin expression and markedly impaired muscle force generation in severe nemaline myopathy. Skelet Muscle. 2011;1(1):23 10.1186/2044-5040-1-23 21798101 PMC3156646

[10] Lehtokari VL, Kiiski K, Sandaradura SA, Laporte J, Repo P, Frey JA, et al Mutation update: the spectra of nebulin variants and associated myopathies. Hum Mutat. 2014;35(12):1418–26. 10.1002/humu.22693 25205138 PMC4295925

[11] Wallgren-Pettersson C, Sewry CA, Nowak KJ, Laing NG. Nemaline myopathies. Semin Pediatr Neurol. 2011;18(4):230–8. 10.1016/j.spen.2011.10.004 . 22172418

[12] Ottenheijm CA, Hooijman P, DeChene ET, Stienen GJ, Beggs AH, Granzier H. Altered myofilament function depresses force generation in patients with nebulin-based nemaline myopathy (NEM2). J Struct Biol. 2010;170(2):334–43. 10.1016/j.jsb.2009.11.013 19944167 PMC2856782

[13] Malfatti E, Lehtokari VL, Bohm J, De Winter JM, Schaffer U, Estournet B, et al Muscle histopathology in nebulin-related nemaline myopathy: ultrastrastructural findings correlated to disease severity and genotype. Acta Neuropathol Commun. 2014;2:44 10.1186/2051-5960-2-44 24725366 PMC4234932

[14] Ryan MM, Schnell C, Strickland CD, Shield LK, Morgan G, Iannaccone ST, et al Nemaline myopathy: a clinical study of 143 cases. Ann Neurol. 2001;50(3):312–20. 10.1002/ana.1080 . 11558787

[15] Pelin K, Hilpela P, Donner K, Sewry C, Akkari PA, Wilton SD, et al Mutations in the nebulin gene associated with autosomal recessive nemaline myopathy. Proc Natl Acad Sci U S A. 1999;96(5):2305–10. 10.1073/pnas.96.5.2305 10051637 PMC26779

[16] Wallgren-Pettersson C, Pelin K, Nowak KJ, Muntoni F, Romero NB, Goebel HH, et al Genotype-phenotype correlations in nemaline myopathy caused by mutations in the genes for nebulin and skeletal muscle alpha-actin. Neuromuscul Disord. 2004;14(8–9):461–70. 10.1016/j.nmd.2004.03.006 . 15336686

[17] Wang CH, Dowling JJ, North K, Schroth MK, Sejersen T, Shapiro F, et al Consensus statement on standard of care for congenital myopathies. J Child Neurol. 2012;27(3):363–82. 10.1177/0883073812436605 22431881 PMC5234865

[18] Teerlink JR, Clarke CP, Saikali KG, Lee JH, Chen MM, Escandon RD, et al Dose-dependent augmentation of cardiac systolic function with the selective cardiac myosin activator, omecamtiv mecarbil: a first-in-man study. Lancet. 2011;378(9792):667–75. 10.1016/S0140-6736(11)61219-1 . 21856480

[19] Psotka MA, Teerlink JR. Direct Myosin Activation by Omecamtiv Mecarbil for Heart Failure with Reduced Ejection Fraction. Handb Exp Pharmacol. 2017;243:465–90. 10.1007/164_2017_13 . 28315072

[20] Woody MS, Greenberg MJ, Barua B, Winkelmann DA, Goldman YE, Ostap EM. Positive cardiac inotrope omecamtiv mecarbil activates muscle despite suppressing the myosin working stroke. Nat Commun. 2018;9(1):3838 10.1038/s41467-018-06193-2 30242219 PMC6155018

[21] Schiaffino S, Reggiani C. Molecular diversity of myofibrillar proteins: gene regulation and functional significance. Physiol Rev. 1996;76(2):371–423. 10.1152/physrev.1996.76.2.371 . 8618961

[22] Schiaffino S, Reggiani C. Fiber types in mammalian skeletal muscles. Physiol Rev. 2011;91(4):1447–531. 10.1152/physrev.00031.2010 . 22013216

[23] Tirrell TF, Cook MS, Carr JA, Lin E, Ward SR, Lieber RL. Human skeletal muscle biochemical diversity. J Exp Biol. 2012;215(Pt 15):2551–9. 10.1242/jeb.069385 22786631 PMC3394665

[24] Hitomi Y, Kizaki T, Watanabe S, Matsumura G, Fujioka Y, Haga S, et al Seven skeletal muscles rich in slow muscle fibers may function to sustain neutral position in the rodent hindlimb. Comp Biochem Physiol B Biochem Mol Biol. 2005;140(1):45–50. 10.1016/j.cbpc.2004.09.021 . 15621508

[25] Li F, Buck D, De Winter J, Kolb J, Meng H, Birch C, et al Nebulin deficiency in adult muscle causes sarcomere defects and muscle-type-dependent changes in trophicity: novel insights in nemaline myopathy. Hum Mol Genet. 2015;24(18):5219–33. 10.1093/hmg/ddv243 26123491 PMC4550825

[26] Joureau B, de Winter JM, Stam K, Granzier H, Ottenheijm CA. Muscle weakness in respiratory and peripheral skeletal muscles in a mouse model for nebulin-based nemaline myopathy. Neuromuscul Disord. 2017;27(1):83–9. 10.1016/j.nmd.2016.10.004 . 27890461 PMC7392587

[27] Mamidi R, Li J, Gresham KS, Verma S, Doh CY, Li A, et al Dose-Dependent Effects of the Myosin Activator Omecamtiv Mecarbil on Cross-Bridge Behavior and Force Generation in Failing Human Myocardium. Circ Heart Fail. 2017;10(10). 10.1161/CIRCHEARTFAILURE.117.004257 29030372 PMC5685665

[28] Swenson AM, Tang W, Blair CA, Fetrow CM, Unrath WC, Previs MJ, et al Omecamtiv Mecarbil Enhances the Duty Ratio of Human beta-Cardiac Myosin Resulting in Increased Calcium Sensitivity and Slowed Force Development in Cardiac Muscle. J Biol Chem. 2017;292(9):3768–78. 10.1074/jbc.M116.748780 28082673 PMC5339759

[29] Cleland JG, Teerlink JR, Senior R, Nifontov EM, Mc Murray JJ, Lang CC, et al The effects of the cardiac myosin activator, omecamtiv mecarbil, on cardiac function in systolic heart failure: a double-blind, placebo-controlled, crossover, dose-ranging phase 2 trial. Lancet. 2011;378(9792):676–83. 10.1016/S0140-6736(11)61126-4 . 21856481

[30] Teerlink JR, Felker GM, McMurray JJV, Ponikowski P, Metra M, Filippatos GS, et al Acute Treatment With Omecamtiv Mecarbil to Increase Contractility in Acute Heart Failure: The ATOMIC-AHF Study. J Am Coll Cardiol. 2016;67(12):1444–55. 10.1016/j.jacc.2016.01.031 . 27012405

[31] Fukuda N, Wu Y, Farman G, Irving TC, Granzier H. Titin-based modulation of active tension and interfilament lattice spacing in skinned rat cardiac muscle. Pflugers Arch. 2005;449(5):449–57. 10.1007/s00424-004-1354-6 . 15688246

[32] Ogut O, Granzier H, Jin JP. Acidic and basic troponin T isoforms in mature fast-twitch skeletal muscle and effect on contractility. Am J Physiol. 1999;276(5):C1162–70. 10.1152/ajpcell.1999.276.5.C1162 . 10329966

[33] Chung CS, Hutchinson KR, Methawasin M, Saripalli C, Smith JE 3rd, Hidalgo CG, et al Shortening of the elastic tandem immunoglobulin segment of titin leads to diastolic dysfunction. Circulation. 2013;128(1):19–28. 10.1161/CIRCULATIONAHA.112.001268 23709671 PMC3822017

[34] Brenner B. The cross-bridge cycle in muscle. Mechanical, biochemical, and structural studies on single skinned rabbit psoas fibers to characterize cross-bridge kinetics in muscle for correlation with the actomyosin-ATPase in solution. Basic Res Cardiol. 1986;81 Suppl 1:1–15. 10.1007/978-3-662-11374-5_1 . 2947559

[35] Ottenheijm CA, Hidalgo C, Rost K, Gotthardt M, Granzier H. Altered contractility of skeletal muscle in mice deficient in titin’s M-band region. J Mol Biol. 2009;393(1):10–26. 10.1016/j.jmb.2009.08.009 19683008 PMC3419142

[36] Burkholder TJ, Fingado B, Baron S, Lieber RL. Relationship between muscle fiber types and sizes and muscle architectural properties in the mouse hindlimb. J Morphol. 1994;221(2):177–90. 10.1002/jmor.1052210207 . 7932768

[37] Claflin DR, Faulkner JA. Shortening velocity extrapolated to zero load and unloaded shortening velocity of whole rat skeletal muscle. J Physiol. 1985;359:357–63. 10.1113/jphysiol.1985.sp015589 3999042 PMC1193379

[38] Wang L, Kawai M. A re-interpretation of the rate of tension redevelopment (k(TR)) in active muscle. J Muscle Res Cell Motil. 2013;34(5–6):407–15. 10.1007/s10974-013-9366-5 24162314 PMC3909470

[39] Kaplinsky E, Mallarkey G. Cardiac myosin activators for heart failure therapy: focus on omecamtiv mecarbil. Drugs Context. 2018;7:212518 10.7573/dic.212518 29707029 PMC5916097

[40] Liu Y, White HD, Belknap B, Winkelmann DA, Forgacs E. Omecamtiv Mecarbil modulates the kinetic and motile properties of porcine beta-cardiac myosin. Biochemistry. 2015;54(10):1963–75. 10.1021/bi5015166 . 25680381

[41] Gollapudi SK, Reda SM, Chandra M. Omecamtiv Mecarbil Abolishes Length-Mediated Increase in Guinea Pig Cardiac Myofiber Ca(2+) Sensitivity. Biophys J. 2017;113(4):880–8. 10.1016/j.bpj.2017.07.002 28834724 PMC5567428

[42] Nagy L, Kovacs A, Bodi B, Pasztor ET, Fulop GA, Toth A, et al The novel cardiac myosin activator omecamtiv mecarbil increases the calcium sensitivity of force production in isolated cardiomyocytes and skeletal muscle fibres of the rat. Br J Pharmacol. 2015 10.1111/bph.13235 26140433 PMC4562511

[43] Kampourakis T, Zhang X, Sun YB, Irving M. Omecamtiv mercabil and blebbistatin modulate cardiac contractility by perturbing the regulatory state of the myosin filament. J Physiol. 2018;596(1):31–46. 10.1113/JP275050 29052230 PMC5746517

[44] Piazzesi G, Caremani M, Linari M, Reconditi M, Lombardi V. Thick Filament Mechano-Sensing in Skeletal and Cardiac Muscles: A Common Mechanism Able to Adapt the Energetic Cost of the Contraction to the Task. Front Physiol. 2018;9:736 10.3389/fphys.2018.00736 29962967 PMC6010558

[45] Fusi L, Brunello E, Yan Z, Irving M. Thick filament mechano-sensing is a calcium-independent regulatory mechanism in skeletal muscle. Nat Commun. 2016;7:13281 10.1038/ncomms13281 27796302 PMC5095582

[46] Woodhead JL, Zhao FQ, Craig R, Egelman EH, Alamo L, Padron R. Atomic model of a myosin filament in the relaxed state. Nature. 2005;436(7054):1195–9. 10.1038/nature03920 . 16121187

[47] Utter MS, Ryba DM, Li BH, Wolska BM, Solaro RJ. Omecamtiv Mecarbil, a Cardiac Myosin Activator, Increases Ca2+ Sensitivity in Myofilaments With a Dilated Cardiomyopathy Mutant Tropomyosin E54K. J Cardiovasc Pharmacol. 2015;66(4):347–53. 10.1097/FJC.0000000000000286 26065842 PMC5460532

[48] Kolb J, Li F, Methawasin M, Adler M, Escobar YN, Nedrud J, et al Thin filament length in the cardiac sarcomere varies with sarcomere length but is independent of titin and nebulin. J Mol Cell Cardiol. 2016;97:286–94. 10.1016/j.yjmcc.2016.04.013 27139341 PMC5584371

[49] Sieck GC. Design principles for life. Physiology (Bethesda). 2013;28(1):7–8. 10.1152/physiol.00049.2012 . 23280352

[50] Sieck GC, Ferreira LF, Reid MB, Mantilla CB. Mechanical properties of respiratory muscles. Compr Physiol. 2013;3(4):1553–67. 10.1002/cphy.c130003 24265238 PMC3977503

[51] Kiss B, Lee EJ, Ma W, Li FW, Tonino P, Mijailovich SM, et al Nebulin stiffens the thin filament and augments cross-bridge interaction in skeletal muscle. Proc Natl Acad Sci U S A. 2018;115(41):10369–74. 10.1073/pnas.1804726115 30249654 PMC6187167

[52] Zhang X, Kampourakis T, Yan Z, Sevrieva I, Irving M, Sun YB. Distinct contributions of the thin and thick filaments to length-dependent activation in heart muscle. Elife. 2017;6 10.7554/eLife.24081 28229860 PMC5365314

[53] Collibee SE, Bergnes G, Muci A, Browne WFt, Garard M, Hinken AC, et al Discovery of Tirasemtiv, the First Direct Fast Skeletal Muscle Troponin Activator. ACS Med Chem Lett. 2018;9(4):354–8. 10.1021/acsmedchemlett.7b00546 29670700 PMC5900333

[54] Lee EJ, De Winter JM, Buck D, Jasper JR, Malik FI, Labeit S, et al Fast skeletal muscle troponin activation increases force of mouse fast skeletal muscle and ameliorates weakness due to nebulin-deficiency. PLoS One. 2013;8(2):e55861 10.1371/journal.pone.0055861 23437068 PMC3577798

[55] Ottenheijm CA, Buck D, de Winter JM, Ferrara C, Piroddi N, Tesi C, et al Deleting exon 55 from the nebulin gene induces severe muscle weakness in a mouse model for nemaline myopathy. Brain. 2013;136(Pt 6):1718–31. 10.1093/brain/awt113 23715096 PMC3673460

