# Supplementary figures and images for: Omecamtiv mecarbil lowers the contractile deficit in a mouse model of nebulin-based nemaline myopathy
Source: PLoS One. 2019 Nov 13;14(11):e0224467. doi: 10.1371/journal.pone.0224467 (PMC6853306; doi:10.1371/journal.pone.0224467)

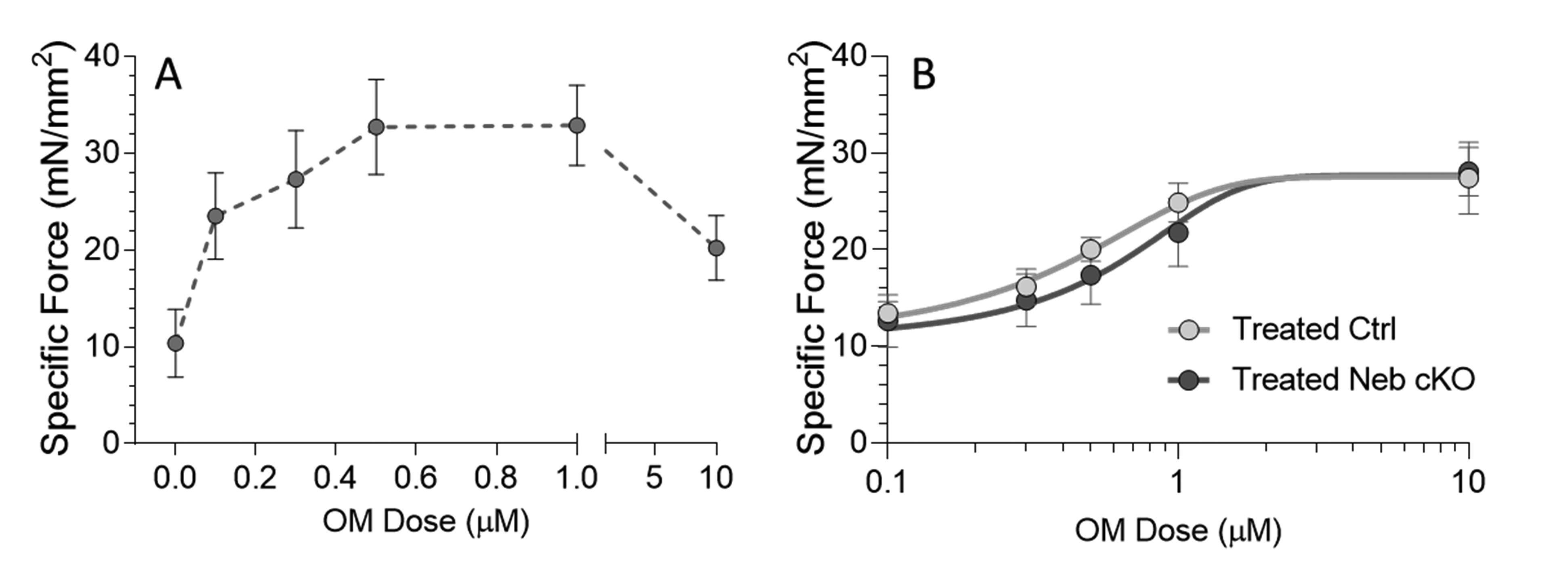

Supplement: S1 Fig — A) Dose-response of OM effect on force production by soleus fiber bundles from Neb cKO mice (n = 6). Specific force was measured at pCa 6.75. OM increases specific force at low OM doses, the effect is maximal between 0.5 and 1.0 μM OM, and is much less at 10 μM OM. B) OM Dose-response using LV permeabilized papillary muscle from control (n = 7) and Neb cKO mice (n = 7). Specific force was measured at pCa 6.0. Specific force follows a dose-response curve with EC50 of 0.62±0.04 μM OM (control) and 0.79±0.08 μM OM (Neb cKO). No significant difference in EC50 (See text for details). (TIF) [file pone.0224467.s001.tif]
